# The Association between Milk and Dairy Products Consumption and Nutrient Intake Adequacy among Japanese Adults: Analysis of the 2016 National Health and Nutrition Survey

**DOI:** 10.3390/nu11102361

**Published:** 2019-10-03

**Authors:** Aki Saito, Emiko Okada, Iori Tarui, Mai Matsumoto, Hidemi Takimoto

**Affiliations:** Department of Nutritional Epidemiology and Shokuiku, National Institutes of Biomedical Innovation, Health and Nutrition, 1-23-1 Toyama Shinjuku-ku, Tokyo 162-8636, Japan; saitoa@nibiohn.go.jp (A.S.); okadae@nibiohn.go.jp (E.O.); nhtun@nibiohn.go.jp (I.T.); m-matsumoto@nibiohn.go.jp (M.M.)

**Keywords:** National Health and Nutrition Survey (NHNS), nutritional adequacy, milk, dairy, Japan

## Abstract

Consumption of dairy products in the usual diet may be important for improving the overall quality of dietary intake. This study aimed to assess the difference in nutrient intake adequacy according to the intake of dairy products based on a 1-day weighed dietary record of Japanese adults from the 2016 National Health and Nutrition Survey. Nutritional adequacy was determined based on the Dietary Reference Intakes for Japanese 2015, with 2 goals: Tentative dietary goals (DG) for preventing lifestyle-related diseases, and the estimated average requirement (EAR). According to the dairy products consumption, participants were categorized into three groups (milk, other dairy product, or non-dairy), and the total number of those not meeting DG and EAR was compared. Non-dairy consumers were less likely to meet both DG and EAR compared to dairy consumers. Dairy consumers were more likely to exceed the DG for saturated fat than non-dairy consumers. Japanese adult dairy consumers were more likely to have adequate nutritional intake than non-dairy consumers, especially for calcium. We also observed a higher saturated fat intake in dairy consumers, which might be due to a certain dietary pattern in this group. Further investigation is needed to determine dairy intake and its influence on dietary quality among the Japanese population.

## 1. Introduction

Dairy products, such as milk, cheese, and yoghurt provide an abundant source of nutrients, including protein, vitamins, and minerals, and their nutritional value has been well recognised [1,2,3]. Their beneficial roles have been examined in association with a variety of chronic diseases, including hypertension, metabolic syndrome, type 2 diabetes, and cardiovascular diseases, as well as in maintaining bone health [3,4,5,6,7,8,9].

Several studies have shown that the intake of dairy products may improve nutrient intake [10,11,12,13,14,15,16]. For example, in a study of Australian children, drinking milk was related to higher intake of micronutrients [10]. Other studies targeting children also reported the positive influence of dairy consumption on the intake of nutrients [11,12,13]. In an Irish study, it also has been suggested that dairy products contribute to the intake of micronutrients such as calcium and retinol [14], while another study has reported that yogurt intake was associated with better diet quality in American adults [15]. Moreover, some researchers have indicated that it is challenging to achieve adequate intake of essential nutrients, such as calcium, potassium, and magnesium, without dairy products in the diet [17]. Studies on dairy intake in relation to nutritional quality have been conducted among Western populations. On the other hand, few comparable studies in Asian countries have been reported. A traditional, typical Japanese diet is a combination of rice, soybean products, fish, seaweed, and green tea [18], which is different from Western countries. The intake of dairy product (milk, cheese, yogurt, and cream) in Japan has been reported to be approximately 110 g/day/capita, including only 63 g/day/capita of milk [18], which is relatively low compared with Western countries [19]. Thus, the influence of the amount/pattern of dairy product consumption on other dietary intakes may differ between Asian and Western populations. Hence, determining the influence of dairy products on adequate nutrient intake among Japanese people using a nationally representative sample is important to Asian populations.

The objective of the present study was to compare nutrient intake adequacy, as well as other food intake, between Japanese adult non-dairy-product consumers, dairy product consumers, and milk consumers, using the 1-day dietary record in the 2016 National Health and Nutrition Survey in Japan (NHNS).

## 2. Materials and Methods

### 2.1. Data Source and Study Population

This study was based on data from the 2016 NHNS. The NHNS is a series of nationally representative, cross-sectional nutrition surveys conducted by local public health centres under the supervision of the Ministry of Health, Labour, and Welfare. The NHNS collects information on health status, food and nutrient intake, and lifestyle of Japanese civilians using a 2-stage cluster sampling scheme. Detailed descriptions of the survey procedures have previously been published [18]. In brief, participants are the individuals aged 1 year or older in the households in selected 475 census units. The survey was conducted from 1 October to 30 November 2016. A total of 10,745 of 24,187 eligible households (44.4%) agreed to participate in the survey. The current study was limited to data collected from 21,851 adults (20 years old and older) who completed information on dietary intake out of 26,225 adult participants. Lactating or pregnant women were excluded from the present analysis since they were presumably not following their usual diet (*n* = 245), for example, due to fear of excess weight gain [20]. Additionally, a previous Japanese study showed that lactating women consumed more dairy products than non-lactating women [21]. The final analytic sample comprised 21,606 participants (9987 men and 11,619 women). This survey was conducted according to the guidelines laid down in the Declaration of Helsinki, and verbal informed consent was obtained from each participant. Under the Statics Act, the Ministry of Health, Labour, and Welfare anonymised individual-level data collected from the NHNS and provided the first author with the datasets for this study. In accordance with the Ethical Guidelines of Epidemiological Research established by the Ministry of Education, Culture, Sports, Science, and Technology, as well as the Ministry of Health, Labour, and Welfare, institutional review board approval was not required for this analysis. As this study is a secondary analysis of the 2016 NHNS, we did not perform a sample size calculation.

### 2.2. Dietary Assessment

Dietary intake data were collected using a 1-day semi-weighed household dietary record. Participants recorded the food consumption of their household for a usual day, excluding trip or festivity days. During the orientation session before the survey, trained fieldworkers (mainly registered dietitians) gave instructions to participants about the survey purpose and how to complete the dietary record. The main record-keepers in a household (members who are usually responsible for preparing meals) were instructed to weigh all foods and beverages consumed by household members and record their names and weights on open-ended recording forms, including the amounts of food waste and leftovers. The main record-keepers were also asked to record the approximate proportions of food consumed by each household member when members shared foods from the same dish, so that the dietary intake of each individual could be calculated. When weighing was not possible, the main-record keepers were asked to record as much information as possible, including portion size consumed or quantity of foods (using household measures) and details of any leftovers. Trained fieldworkers visited each household and checked the completeness of recording forms and, if necessary, confirmed portion sizes using commercially available food models or food booklets, and corrected any missing and/or illogical information. In accordance with the survey manual of the NHNS, the trained fieldworkers converted these estimates of portion sizes or quantities of food into food weights and coded each food item according to the NHNS food number list. These food number lists were based on the Standard Tables of Food Composition in Japan [22]. When the reporting of the ingredients of mixed dishes was uncertain, such as when eating out in restaurants, common food compositions were allocated to the mixed dish according to typical Japanese recipes [21]. The collected recording forms and converted data were further checked at the local public health centre, and trained fieldworkers inputted dietary intake data using software specifically developed for the NHNS. These data were then compiled by trained investigators at the central office to create a whole dietary dataset. Nutrient intake from dietary supplements or nutrient-fortified food were not assessed because of the lack of reliable databases in Japan.

This household-based dietary record method has been compared to individual dietary records in Japanese participants [23]. Intakes in 32 female dietetic students, as recorded by the students themselves, were compared against the 1-day household dietary record completed by their mothers. Pearson correlation coefficients were 0.90 for energy, 0.89 for protein, 0.91 for total fat, and 0.90 for carbohydrate. Additionally, Murakami et al. reported that the energy intake estimates using NHNS were 0.98 times that of the estimated energy requirement (EER) for adults aged 20 years and older [24].

To enable the comparison between the nutrient intake and the Japanese dietary reference intake (DRI) values, we adjusted the reported dietary intake based on the assumption that the energy intake of each participant was the same as his/her EER using the following calculation: dietary intake (unit/day) = reported dietary intake (unit/day)/reported energy intake (kcal/day) × EER (kcal/day). Since we could not obtain data on each participant’s physical activity level, the EER value was determined assuming that each individual’s physical activity level was at the “second” (middle) level in the Japanese DRIs [25]. This level corresponds to a moderate physical activity status where individuals spend most of the day sedentary at work, but including movement and housework, such as commuting and shopping, as well as sports with light intensity [25]. For protein, total fat, saturated fat, and carbohydrate intake, a percentage of the daily energy intake was calculated using the crude values and used for comparison with the DRI values.

### 2.3. Other Variables

Body height (to the nearest 0.1 cm) and weight (to the nearest 0.1 kg) were measured for approximately 90% of the participants by trained field workers using standardised procedures. For the remaining participants, height and weight were measured either by other household members at home or were self-reported. Body mass index (BMI) was calculated as weight (kg) divided by height (m) squared. Using a self-administered questionnaire, smoking status and alcohol drinking habits during the preceding month were assessed.

### 2.4. Determination of Nutritional Intake Adequacy

Adequacy of each nutrient intake was determined by comparing nutrient levels with each dietary reference value according to the Japanese DRIs, using a cut-point method as previously reported [26,27,28]. In the Japanese DRIs [25], different reference values are established according to their purpose. The estimated average requirement (EAR) is an intake value estimated as having a 50% risk of insufficiency and is set to avoid insufficient intake. The tentative goal for preventing lifestyle-related disease (DG) is either an upper or lower limit of the intake value set to prevent lifestyle-related disease. An intake level below the EAR was considered as “not meeting” using the cut-point method for 14 nutrients, which were protein, vitamin A expressed as retinol equivalents, thiamine, riboflavin, niacin expressed as niacin equivalent, vitamin B-6, vitamin B-12, folate, vitamin C, calcium, magnesium, iron (except for women aged 20–49 years), zinc, and copper. Retinol equivalents were calculated as follows: 1 μgRE = retinol (μg) + beta-carotene (μg) × 1/12 + alpha-carotene (μg) × 1/24 + beta-cryptoxantin (μg) × 1/24 + other provitamin A carotenoides (μg) × 1/24. Niacin equivalent was calculated as follows: niacin (mg) + protein (mg)/6000. For iron, the cut-point method is not appropriate for menstruating women [29,30]. Therefore, we applied the value <9.3 mg/day as shown by the World Health Organization (WHO) (bioavailability of iron as 15%, probability of inadequacy as 50%) [31] for women 20–49 years old. Although the EAR was set for biotin, chromium, molybdenum, selenium, and iodine in the Japanese DRIs, these nutrients were excluded from this study because food composition tables in Japan provide incomplete data for these nutrients. An intake level falling outside the range of DG values was considered as not meeting the standard for 7 nutrients of the DG, which were percent energy from protein, total fat, saturated fat, carbohydrates, total dietary fibre, sodium, and potassium. Sodium was calculated as salt-equivalent (salt (g) = 58.5/23 × sodium (g)).

### 2.5. Statistical Analysis

All statistical analyses were performed with SAS statistical software, version 9.4 (SAS Institute Inc., Cary, NC, USA). All reported *p* values were two-tailed, with a *p* value < 0.05 considered statistically significant. All statistical analyses were performed separately by sex.

The participants were categorized into 3 groups according to their intake of milk and dairy products: Those who reported consuming liquid milk (cow milk: full-fat, low-fat, non-fat milk, and flavoured milk) with or without other dairy products (whole milk powder, cream, yoghurt, natural cheese, and processed cheese) were categorized as “milk consumers”; those who reported consuming other dairy products but not milk were categorized as “other dairy consumers”; and those who did not consume any milk nor dairy products were categorized as “non-dairy consumers”. Table 1 shows the definition of milk and dairy products in this study. The mean of age and BMI (continuous variables) of the 3 groups (independent variable with fixed effect) was compared using an analysis of variance (ANOVA) with SAS PROC ANOVA command. The chi-square test was used to compare proportions of age category, BMI category, residential block, current smokers, and habitual drinkers (categorical variables) using the PROC FREQ command. We also examined the intake of different groups of food based on the NHNS food group categories [18], presented by intake weight (grams) per energy intake of 1000 kcal, across the participant groups, using the Kruskal–Wallis test (PROC NPAR1WAY).

The median and interquartile range (IQR) of energy and nutrient intake were calculated for each group and significant differences across the groups were assessed using the Kruskal–Wallis test and subsequent post hoc analyses (Dwass, Steek, and Critchlow–Fligner method). The percentage of participants whose intake was below the EAR or outside the range of the DG was estimated. The Cochran—Mantel—Haenszel test was used to examine the difference in the preference of not meeting DRIs between the groups adjusting for age categories (PROC FREQ). To examine the overall nutritional adequacy of each participant, the number of nutrients with EAR and DG was counted, respectively. The number of nutrients with not meeting DRIs was 0–14 for the EAR and 0–7 for the DG. The difference in the numbers of nutrients with not meeting DRIs between the groups was assessed using analysis of co-variance (ANCOVA) adjusting for the age categories (PROC GLM).

## 3. Results

The basic characteristics of the participants according to dairy product consumption are shown in Table 2. The mean ages were 57.2 years (standard deviation (SD), 17.4) old for men and 58.8 years old for women (SD, 17.5). The mean milk intake was 60.9 g/day (SD, 109.6) and 69.5 g/day (SD, 107.2) and that of other dairy products was 32.8 g/day (SD, 60.5) and 43.7 g/day (SD, 64.8) in men and women, respectively. A total of 3990 men (40.0%) and 5345 women (46.0%) reported consuming liquid milk and 2364 men (23.7%) and 3102 women (26.7%) reported consuming other dairy products on the dietary recording day, while the others did not consume dairy products. There were significant differences in the mean age, BMI, proportions of each BMI category, residential block, and the number of current smokers and habitual alcohol drinkers across the groups in both men and women (*p* < 0.05).

Table 3 shows food group intake across the dairy consumption status. Men and women showed similar differences in food intakes across the groups. In both sexes, the intake of “grains”, “sugars”, “sesame and nuts”, “vegetables”, “fruits”, “mushrooms”, “fish and shellfish”, “oils”, “confectionaries”, “beverages”, and “seasonings” were significantly different (*p* < 0.05 for each food group). In men, “pulses” and “egg” intake were also significantly different among the groups (*p* = 0.037, *p* = 0.002, respectively), while “seaweeds” intake was significantly different among the groups in women (*p* = 0.029). Non-dairy consumers had the highest intakes of “grains” and “seasonings”, while they also had the lowest intakes of “sugars”, “sesame and nuts”, “fruits”, “mushrooms”, “oils”, and “confectionaries” (*p* < 0.05). Among men, non-dairy consumers also had the lowest intake of “vegetables” (*p* < 0.05). In milk consumers, the intake of the “oil” food group was higher than in other dairy consumers and non-dairy consumers in both men and women, while the intake of “fish and shellfish” was lower than in the other two groups (*p* < 0.05). Other dairy consumers had a higher intake of “beverages” than the other two groups (*p* < 0.05).

Table 4 and Table 5 show the energy and nutrient intake and prevalence of not meeting DG and EAR across the groups in men and women, respectively. Significant differences in mean energy and nutrient intake values between the groups were observed for all nutrients in men and women (*p* < 0.05), except for vitamin B12 in men. In men, carbohydrate and sodium intakes were higher among non-dairy consumers, while all the other nutrient intakes, except for niacin and iron, were higher in milk and/or other dairy consumers. In women, carbohydrate, sodium, and copper intakes were higher among non-dairy consumers, while dietary fibre, folate, and vitamin C intakes were the highest in other dairy consumers (*p* < 0.05). Iron intake was lower among milk consumers compared to other groups (*p* < 0.05). Intake of all other nutrients, except for niacin, in milk and/or other dairy consumers compared to non-dairy consumers was higher in women. Among men, the prevalence of not meeting DRIs was only significantly higher for saturated fat in milk consumers and dairy consumers than in non-dairy consumers. The prevalence of not meeting DRIs for all remaining nutrients, except for niacin and copper, was significantly higher among non-dairy consumers than other groups (*p* < 0.05). Among women, saturated fat was the only nutrient that showed a higher prevalence of not meeting DRIs in milk or other dairy consumers than in non-dairy consumers (*p* < 0.001). The prevalence of not meeting DRIs for the other nutrients, except for total fat, dietary fibre and copper, was higher in non-dairy consumers (*p* < 0.05).

The mean numbers of nutrients with not meeting DG and EAR are shown in Table 6. Among men, the mean number of nutrients with not meeting DG was 3.8 for milk consumers, 3.8 for other dairy consumers, and 4.0 for non-dairy consumers. For not meeting EAR, the mean number was 3.3 for milk consumers, 3.9 for other dairy consumers, and 4.7 for non-dairy consumers. After age-adjustment, non-dairy consumers had a significantly higher number of nutrients with not meeting DG and EAR than other groups (*p* < 0.001). Among women, the mean number of nutrients with not meeting DG also showed a significant difference across the groups, with the numbers of nutrients calculated at 3.8 in the milk consumers, 3.7 for other dairy consumers, and 3.9 for non-dairy consumers (*p* = 0.02). The number of nutrients with not meeting EAR was 3.3, 3.9, and 4.7 in milk consumers, other dairy consumers, and non-dairy consumers, respectively (*p* < 0.001).

## 4. Discussion

In the present study, we compared the nutrient intake adequacy of individuals who reported consuming milk or other dairy products to that of those who reported not consuming any dairy products using a 1-day dietary record. In a comparison of the number of nutrients not meeting DRIs, dairy consumers (both milk consumers and other dairy consumers) were more likely to have adequate nutrient intake than non-dairy consumers. However, for saturated fat, dairy consumers had higher intake (above the DG level) than non-dairy consumers. To the best of our knowledge, this is the first report that studied nutritional adequacy according to milk and dairy products intake among Japanese adults. It is important to examine this issue in a population with lower dairy intake to understand the influence of dairy products intake on nutritional intake in people with different characteristics.

Even among the dairy consumers in the current study, the mean daily intake of dairy products was approximately 160 g in milk consumers and 70 g in other dairy consumers; this was the same in both men and women. In an Irish study, the mean dairy product intake in dairy consumers was reported as 291 g [14], and the average liquid milk intake in the US was reported to be around 180 mL (186 g, using 103.2 g/100 mL for conversion factor [32]) [33], while in Japan the reported amount is about 60 mL (62 g) [18]. In the current study, dairy consumers had less intake of “grains” and “seasoning” than non-dairy consumers. In addition, milk consumers had less intakes of “fish and shellfish” than non-dairy consumers. A previous study has indicated that dairy products are consumed with bread rather than rice or noodles as dietary staples among Japanese population [34]. Together with the present findings, this suggests that a non-dairy diet may be a result of rice-based meals with greater amounts of fish, which is regarded as a traditional Japanese diet. In turn, this might also suggest that a diet with dairy products is a Westernised Japanese diet. Another Japanese study using NHNS data described that a dietary pattern mainly consisting of bread and dairy products has been increasing in Japanese adults [35], which may point to a shift in the Japanese diet, despite much lower dairy consumption compared to that in Western countries.

The association between dairy products intake and nutritional intake has been examined in several studies, mainly in Western countries. For example, a diet modelling study showed that increasing dairy food consumption to recommended amounts could improve the intake of essential vitamins and minerals that were inadequately consumed [36]. Similar to our results, a US study reported that consumption of milk (plain or flavoured) was associated with better macro- and micronutrients intake among children and adolescents [11], and other studies have shown similar results [6,10]. In the present study, the greatest difference in inadequate intake of nutrients between milk consumers and non-dairy consumers was found to be for calcium, which could be greatly responsible for the total number difference of those not meeting EAR. The contribution of dairy intake to calcium intake has been reported using nutrient profiling methods [37]. Only 28% of the calcium intake of a Japanese adult is received from dairy products. Other foods, especially vegetables or pulses, also contribute to calcium intake in the Japanese diet [18]. Although in this study the intake of “vegetables” was higher in dairy consuming men, a similar pattern was not observed in the intakes of “pulses” and “vegetables” in dairy consuming women. Therefore, dairy intake itself might be responsible for the relatively adequate calcium intake in this study. However, there still was a considerable number of non-dairy consumers (approximately 20%) who achieved adequate calcium intake without dairy products. This is not consistent with previous Western studies which suggested that milk and dairy intake particularly influence calcium intake because only those who consumed milk had an average dietary calcium intake close to their recommended levels [10,11,17,36]. Further studies are needed to explore the factors contributing to adequate calcium intake in the Japanese population for future public health strategies. On another note, more fruit and mushrooms were consumed by dairy consumers in this study. This result is also similar to that described in a previous study, which identified a combination of dairy products and fruit as one of the typical meals among Japanese people [34]. These food intakes among dairy consumers could also explain the better intake of certain nutrients, such as vitamin C and dietary fibre, because milk and many other dairy products lack such nutrients [22]. Our finding also suggests that older people are more likely to consume dairy products, which is consistent with the annual reports of NHNS showing higher dairy products intake among older people than younger people [18]. The NHNS has shown that older people are more likely to have better dietary intake, such as more fruit and vegetables [18]. Further assessments are needed to investigate the dietary intake patterns according to the age difference, and in relation to dairy products intake.

Despite adequate nutritional intake observed among dairy consumers for EAR, the difference in the number of those not meeting DG was small, even though statistically significant differences were observed. The DG values were set to achieve better nutritional intake from the standpoint of prevention of life-style related diseases such as diabetes, hypertension, and dyslipidaemia [25]. Among nutrients with DG, the difference in the prevalence of not meeting DG was observed for several nutrients such as potassium and protein according to dairy intake status, with more favourable intake in dairy consumers. However, there was a higher prevalence of excess saturated fat intake in both male and female dairy consumers. These could substantially explain the small difference in the number of not meeting DG compared to not meeting EAR. Regarding excess saturated fat intake, similar results have been observed in previous studies [10,11,13,14,17]. In NHNS 2016, in the same participants used as the sample for this study, 17% of saturated fat came from dairy products [18]. Dairy product intake has been identified in meals with bread in Japanese adults [34] and another Japanese study reported that breakfast bread intake was associated with higher saturated fat intake in young women [38]. From these findings, not only dairy foods, but the combination with bread may play an important role in Japanese saturated fat intake. According to a Japanese dietary guideline (Japanese Food Guide Spinning Top), daily intake of one serving of milk (200 mL; 206 g) or its equivalent is recommended [39], which contains a saturated fat level of 4.8 g (full-fat milk) [22]. This milk amount exceeds the median milk intake of the participants of the current study, even that of milk consumers. A Japanese study has attempted to design optimal dietary intake to achieve a full set of nutritional recommendations in the Japanese DRIs, suggesting a reduction in consuming full-fat dairy and increasing the intake of low-fat dairy products [40]. This might partly reflect the contribution of full-fat dairy to saturated fat intake among Japanese adults. In fact, most of the milk consumed in this study was full-fat milk, with the mean low-fat milk consumption being 21.1 g and 20.6 g in men and women, respectively (data not shown). Therefore, to develop an appropriate public health strategy, further studies using diet modelling methods are needed to set an adequate dairy intake in the Japanese diet. In contrast, a US study has recently reported that long-term intake of dairy fatty acids was not associated with all-cause mortality and cardiovascular disease mortality among older adults [41]. Furthermore, there has been a report showing a negative association between risk of cardiovascular diseases and dairy saturated fat intake [42]. The effectiveness of dairy product intake as a source of saturated fat intake also needs to be further examined.

Several limitations of this study need to be mentioned. First, the study samples were randomly selected from nationally representative households in Japan; however, only 44.4% of selected households participated in the survey. Furthermore, the individual-level response rate within the household is unknown. Additionally, the dietary survey was conducted on intentionally selected single weekdays in only two months from October to December. This might induce some bias in the estimation of average intake in Japanese adults. Second, a dietary intake derived from a 1-day weighed dietary record is unlikely to represent the usual intake of individual respondents. Therefore, day-to-day variability in the dietary intake of individuals might have influenced the findings. Moreover, while the validity of the household-based dietary record used for this survey has been examined among young women, the true utility of this methodology for assessing daily dietary intake among men or people in other age groups is unknown. Furthermore, measurement error could be caused by misreporting of self-reported food intake. Finally, we classified the participants according to their milk or dairy consumption only, regardless of the amount consumed. Further investigation is needed to estimate the adequate number of dairy products. Additionally, it should be noted that diet quality is defined by the totality of foods consumed rather than by the presence or absence of a single food [43,44,45]. Although nutrition intake was better among dairy consumers in part, public health messages should focus on promoting healthier food choices.

## 5. Conclusions

In conclusion, despite lower dairy intake compared to Western populations, dairy consumers (milk consumers and other dairy consumers) had more adequate nutritional intake, particularly for calcium, than non-dairy consumers. However, a diet with dairy products was more likely to associate with excess saturated fat intake. Although dairy intake might be a sign of healthier dietary behaviour in Japanese adults, further investigation is needed to determine dairy intake and its influence on overall dietary intake among the Japanese population.

## Figures and Tables

**Table 1:** Definition of milk and dairy products in the study.

Food Group	Included Foods	Food Item Number in the Standard Tables of Food Composition in Japan [20]
Milk	Ordinary milk, high fat milk, low fat milk, skimmed milk, milk beverages	13001–13010
Other dairy products	Milk powder, cream, yogurt, cheese	13012–13028, 13031–13041

**Table 2:** Basic characteristics of 9987 men and 11,619 women according to dairy intake status ^1, 2.^

	Men					Women				
	All	Milk	Dairy	Non–Dairy	*p* ^3^	All	Milk	Dairy	Non–Dairy	*p* ^3^
*n*	9987	3990	2364	3633		11619	5345	3102	3172	
Age, year	57.2 ± 17.4	58.3 ± 17.8	58.3 ± 16.7	55.2 ± 17.5	<0.001	58.8 ± 17.5	58.9 ± 17.4	59.7 ± 16.7	57.8 ± 18.5	<0.001
Age category, %					<0.001					<0.001
20–29	7.1	6.7	6.5	8.0		6.1	5.8	5.1	7.8	
30–39	12.1	12.1	9.6	13.7		10.2	9.9	9.2	11.9	
40–49	15.8	14.1	15.1	18.2		15.5	16.3	14.2	15.4	
50–59	14.9	13.9	15.4	15.6		15.3	15.1	16.1	14.8	
60–69	23.1	22.6	25.9	21.9		22.7	22.7	25.3	20.4	
70–	27.0	30.7	27.5	22.7		30.1	30.2	30.2	29.8	
“Milk” intake, g/day	0.0 (0.0–103.0)	157.8 (38.3–206.0)	0 (0–0)	0 (0–0)		0 (0.0–150.0)	154.5 (52.0–206.0)	0 (0–0)	0 (0–0)	
“Other dairy products” intake, g/day	0.0 (0.0–50.0)	2.0 (0.0–70.0)	65.0 (10.0–100.0)	0 (0–0)		0 (0.0–79.0)	15.0 (0.0–83.0)	72.6 (15.0–108.0)	0 (0–0)	
Body mass index, kg/m^2^										
*n*	7517	3131	1808	2578		9271	4413	2489	2369	
	23.8 ± 3.5	23.7 ± 3.4	23.7 ± 3.4	24.0 ± 3.6	<0.001	22.5 ± 3.7	22.4 ± 3.6	22.6 ± 3.6	22.8 ± 4.0	<0.001
Body mass index category, %					0.026					<0.001
Underweight (<18.5)	4.0	4.1	3.7	4.0		10.9	10.8	10.0	12.0	
Normal (18.5–25)	64.1	65.7	64.7	61.8		67.7	69.4	68.5	63.8	
Overweight (25+)	31.9	30.2	31.7	34.2		21.4	19.9	21.5	24.2	
Residential block, %					<0.001					<0.001
Hokkaido and Tohoku	14.9	14.2	14.1	16.3		15.0	14.3	14.0	17.4	
Kanto	22.1	22.5	23.5	20.8		20.8	21.5	20.9	19.4	
Hokuriku and Tokai	19.7	18.2	20.0	21.1		19.4	18.1	20.3	20.7	
Kinki	11.1	11.9	11.6	9.9		11.4	12.4	11.5	9.7	
Shikoku and Chugoku	19.6	21.0	19.6	18.0		20.2	21.3	20.8	17.7	
Kyusyu	12.6	12.2	11.2	13.9		13.2	12.4	12.5	15.3	
Current smoking, %										
*n*	9770	3917	2310	3543		11423	5271	3064	3088	
					<0.001					<0.001
Yes	29.1	24.4	28.4	34.9		7.0	5.4	6.5	10.2	
No	70.9	75.6	71.6	65.1		93.0	94.6	93.5	89.8	
Habitual alcohol drinking, %										
*n*	9756	3909	2308	3539		11408	5269	3058	3081	
					<0.001					<0.001
Yes	34.3	29.8	34.7	39.1		7.5	6.1	8.2	9.4	
No	65.7	70.2	65.3	60.9		92.5	93.9	91.8	90.6	

^1^ Values are mean ± SD, %, or median (IQR). ^2^ Milk, milk consumers; Dairy, other dairy consumers; Non-dairy, non-dairy consumers. ^3^ For continuous variables, ANOVA was used; for categorical variables, a chi-square test was used to test differences across the groups.

**Table 3:** Intakes of selected food groups (g/1000kcal) among 9987 men and 11,619 women according to dairy intake status ^1,2^

Sex	Food Groups (g/1000kcal)	All	Milk	Dairy	Non-Dairy	*p* ^3^
Men						
	Grains	240.6 (192.3–293.2)	223.3 (179.3–270.9)	232.4 (188.2–283.8)	267.2 (214.9–319.7)	<0.001
	Potatoes	16.9 (0.0–41.2)	18.0 (0.0–40.9)	16.3 (0.0–40.9)	15.3 (0.0–41.5)	0.09
	Sugars	2.0 (0.2–4.6)	2.3 (0.5–4.9)	2.4 (0.5–5.1)	1.5 (0.0–3.9)	<0.001
	Pulses	21.0 (0.0–48.0)	21.0 (1.7–45.7)	22.7 (1.4–49.0)	19.8 (0.0–50.1)	0.037
	Sesame and nuts	0.0 (0.0–0.4)	0.0 (0.0–0.6)	0.0 (0.0–0.5)	0.0 (0.0–0.0)	<0.001
	Vegetables	127.1 (81.2–184.1)	130.0 (86.8–184.8)	131.9 (82.9–189.2)	120.3 (73.7–179.2)	<0.001
	Fruits	16.0 (0.0–71.1)	33.3 (0.0–80.8)	32.7 (0.0–79.8)	0.0 (0.0–48.6)	<0.001
	Mushrooms	1.2 (0.0–12.1)	2.2 (0.0–12.2)	2.3 (0.0–13.3)	0.0 (0.0–11.2)	<0.001
	Seaweeds	1.1 (0.0–7.3)	1.1 (0.0–7.5)	1.1 (0.0–7.6)	1.0 (0.0–7.0)	0.08
	Fish and shellfish	33.0 (7.1–58.7)	30.5 (6.9–54.3)	34.5 (7.9–61.5)	34.7 (7.0–62.9)	<0.001
	Meat and poultry	44.9 (22.3–70.6)	44.4 (23.9–68.4)	44.8 (21.1–68.2)	46.0 (20.8–75.2)	0.10
	Eggs	17.4 (1.5–29.4)	17.9 (2.9–28.9)	17.8 (1.3–29.6)	16.2 (0.0–30.1)	0.002
	Oils	4.6 (2.0–7.9)	4.9 (2.4–8.1)	4.6 (2.0–7.8)	4.3 (1.6–7.7)	<0.001
	Confectionaries	0.0 (0.0–11.2)	0.0 (0.0–14.7)	0.0 (0.0–13.3)	0.0 (0.0–3.3)	<0.001
	Beverages	321.4 (177.2–494.7)	301.2 (169.3–459.9)	349.8 (204.7–518.4)	324.5 (167.6–522.9)	<0.001
	Seasonings	33.3 (22.7–56.6)	31.7 (21.9–50.8)	33.7 (22.9–57.0)	35.0 (23.6–63.0)	<0.001
Women						
	Grains	215.9 (168.8–269.3)	201.0 (157.9–248.2)	213.0 (166.9–263.1)	251.4 (200.9–307.8)	<0.001
	Potatoes	20.5 (0.0–47.9)	20.6 (0.0–46.3)	21.0 (0.0–48.5)	20.1 (0.0–51.0)	0.86
	Sugars	2.5 (0.4–5.6)	2.8 (0.7–5.9)	2.8 (0.6–6.2)	1.9 (0.0–4.7)	<0.001
	Pulses	25.8 (1.8–55.9)	25.2 (2.6–52.6)	26.4 (3.1–57.8)	25.9 (0.0–60.8)	0.06
	Sesame and nuts	0.0 (0.0–0.7)	0.0 (0.0–1.2)	0.0 (0.0–0.8)	0.0 (0.0–0.0)	<0.001
	Vegetables	151.0 (97.4–214.1)	150.3 (99.0–208.2)	155.0 (99.6–222.0)	148.8 (92.6–216.4)	0.005
	Fruits	49.0 (0.0–104.8)	56.7 (1.7–107.5)	55.9 (0.0–115.1)	12.6 (0.0–86.0)	<0.001
	Mushrooms	2.8 (0.0–15.1)	3.5 (0.0–15.0)	3.2 (0.0–16.1)	0.0 (0.0–14.1)	<0.001
	Seaweeds	0.9 (0.0–8.2)	1.0 (0.0–8.0)	0.9 (0.0–9.3)	0.6 (0.0–7.6)	0.029
	Fish and shellfish	34.1 (5.9–61.5)	31.8 (5.9–57.0)	36.2 (7.3–64.2)	36.9 (4.4–66.7)	<0.001
	Meat and poultry	40.4 (17.9–65.5)	39.9 (18.8–63.5)	39.7 (16.9–64.5)	42.0 (16.6–69.7)	0.10
	Eggs	18.9 (0.4–32.7)	18.8 (1.7–31.3)	19.1 (0.0–32.6)	18.9 (0.0–35.1)	0.80
	Oils	4.6 (1.9–8.1)	4.8 (2.2–8.3)	4.6 (1.8–8.1)	4.2 (1.5–7.9)	<0.001
	Confectionaries	0.0 (0.0–22.1)	3.5 (0.0–22.8)	0.0 (0.0–23.7)	0.0 (0.0–18.6)	<0.001
	Beverages	322.7 (178.9–496.3)	300.2 (169.5–457.9)	355.5 (215.3–526.0)	324.4 (163.5–528.7)	<0.001
	Seasonings	35.1 (23.7–56.2)	33.6 (22.8–53.5)	35.4 (24.3–56.6)	37.2 (24.8–61.1)	<0.001

^1^ Values are medians (IQR). Food groups are expressed as amount per 1000 kcal energy intake. ^2^ Milk, milk consumers; Dairy, other dairy consumers; Non-dairy, non-dairy consumers. ^3^ The *p* value shows the result of Kruskal–Wallis tests to compare food intakes between dairy intake groups.

**Table 4:** Daily nutrient intakes and prevalence of not meeting DRIs (DG and EAR) among 9987 men according to dairy intake status ^1,2,3,4.^

	All		Milk		Dairy		Non-Dairy			
	Intakes	Not Meeting DRIs: % (above)	Intakes	Not Meeting DRIs: % (above)	Intakes	Not Meeting DRIs: % (above)	Intakes	Not Meeting DRIs: % (above)	*p* ^5^	
Energy, kcal/day	2062.8 (1720.2–2442.8)		2144.9 (1808.2–2517.6)		2083.8 (1764.4–2447.6)		1945.7 (1580.8–2349.3)			
Nutrients with DG										
Protein, % energy	14.3 (12.5–16.3)	36.5	14.6 (12.8–16.4)	31.4	14.4 (12.6–16.4)	35.3	14.0 (11.9–16.2)	42.8	<0.001	
		(4.6)		(4.0)		(5.0)		(5.1)		
Fat, % energy	25.4 (20.6–30.5)	49.3	26.8 (22.2–31.3)	46.0	25.2 (20.4–30.5)	50.0	24.0 (18.9–29.2)	52.4	<0.001	
		(27.1)		(31.3)		(27.3)		(22.3)		
Saturated fat, % energy	6.5 (5.0–8.3)	41.8	7.3 (6.0–9.0)	56.2	6.3 (4.9–8.0)	38.9	5.6 (4.1–7.2)	28.0	<0.001	
Carbohydrate, % energy	59.9 (54.3–65.3)	38.5	58.5 (53.4–63.4)	33.1	60.1 (54.4–65.1)	38.1	61.6 (55.5–67.6)	44.7	<0.001	
		(26.3)		(19.0)		(25.7)		(34.9)		
Dietary fibre, g/d	16.8 (13.0–21.4)	67.0	17.0 (13.4–21.5)	65.7	17.4 (13.5–22.0)	64.9	16.1 (12.3–20.9)	69.8	<0.001	
Sodium (salt-equivalent), g/day	12.2 (9.8–15.2)	89.3	11.7 (9.5–14.3)	88.4	12.2 (9.8–15.3)	89.8	12.8 (10.1–16.1)	89.9	0.08	
Potassium, mg/day	2679 (2193–3246)	65.4	2828 (2358–3366)	58.4	2725 (2243–3305)	63.5	2471 (2015–3018)	74.4	<0.001	
Nutrients with EAR										
Protein, g/d	87.4 (76.1–100.0)	0.9	88.2 (78.0–99.5)	0.4	87.5 (76.5–101.0)	0.8	86.5 (73.2–100.2)	1.7	<0.001	
Vitamin A, μg RE/day	470 (302–708)	65.1	528 (357–755)	58.8	465 (303–700)	65.6	409 (239–639)	71.7	<0.001	
Thiamine, mg/day	1.00 (0.80–1.27)	60.9	1.02 (0.84–1.27)	59.0	1.00 (0.78–1.29)	60.2	0.98 (0.76–1.27)	63.4	<0.001	
Riboflavin, mg/day	1.33 (1.06–1.65)	38.5	1.46 (1.21–1.77)	24.8	1.30 (1.06–1.60)	39.5	1.18 (0.93–1.49)	53.0	<0.001	
Niacin, NEmg/day	32.9 (27.3–39.3)	0.0	32.5 (27.5–38.2)	0.0	33.5 (27.6–40.2)		32.9 (26.8–40.1)	0.1	0.29	
Vitamin B-6, mg/day	1.38 (1.12–1.69)	32.8	1.41 (1.15–1.69)	29.5	1.39 (1.13–1.71)	31.7	1.34 (1.08–1.67)	37.1	<0.001	
Vitamin B-12, μg/day	5.4 (2.9–10.2)	13.6	5.4 (3.1–9.7)	9.3	5.5 (2.9–10.6)	14.4	5.3 (2.6–10.6)	17.9	<0.001	
Folate, μg/day	328 (251–421)	11.0	332 (259–420)	9.0	336 (262–434)	8.9	317 (237–412)	14.5	<0.001	
Vitamin C, mg/day	89.8 (55.6–140.9)	46.8	95.6 (60.2–147.3)	43.1	97.8 (59.6–150.1)	42.1	79.6 (48.2–125.2)	53.8	<0.001	
Calcium, mg/day Magnesium, mg/day	544 (390–738)	56.4	672 (511–850)	37.1	551 (416–719)	56.7	416 (312–565)	77.5	<0.001	
	296 (248–353)	46.1	302 (256–354)	41.8	302 (256–360)	43.0	284 (237–345)	52.9	<0.001	
Iron, mg/day	9.2 (7.6–11.0)	8.3	9.1 (7.5–10.8)	7.6	9.3 (7.7–11.2)	7.1	9.2 (7.5–11.2)	10.0	0.002	
Zinc, mg/day	10.0 (8.8–11.5)	13.6	10.2 (9.0–11.6)	10.8	9.9 (8.7–11.4)	14.6	9.9 (8.6–11.4)	16.0	<0.001	
Copper, mg/day	1.4 (1.2–1.6)	0.6	1.4 (1.2–1.6)	0.4	1.4 (1.2–1.7)	0.5	1.5 (1.2–1.7)	0.8	0.11	

^1^ Intakes values are medians (IQR). Nutrients expressed as energy-adjusted values by using the following equation: energy-adjusted intake (unit/day) = observed intake (unit/day) × Estimated Energy Requirement (EER, kcal/day) / observed energy intake (kcal/day). ^2^ Prevalence of not meeting DRIs is percentage of participants whose intake fell outside the range of dietary goals (DG) or below the estimated average requirement (EAR). Each energy-adjusted nutrient intake (unit/day) was compared with each DRI (dietary reference intake) value (unit/day), using the cut-point method. The probability of inadequacy of >50% for menstruating women whose bioavailability of iron is 15% (<9.3 mg/day) was considered not meeting EAR for women aged 20–49 years. ^3^ All nutrient intakes, except vitamin B12 in men, were significantly different across the dairy intake groups (*p* < 0.05; Kruskal–Wallis test). ^4^ Milk, milk consumers; Dairy, other dairy consumers; Non-dairy, non-dairy consumers; RE, retinol equivalent; NE, niacin equivalent. ^5^ Difference in the prevalence of not meeting DG and EAR was tested using Cochran–Mantel–Haenszel test adjusting for age categories.

**Table 5:** Daily nutrient intakes and prevalence of not meeting DRIs (DG and EAR) among 11,619 women according to dairy intake status ^1,2,3,4.^

	All		Milk		Dairy			Non-Dairy		
	Intakes	Not Meeting DRIs: %(above)	Intakes	Not Meeting DRIs: %(above)		Intakes	Not Meeting DRIs: %(above)	Intakes	Not Meeting DRIs: %(above)	*p* ^5^
Energy, kcal/d	1654.0 (1386.2–1943.8)		1742.0 (1482.8–2014.8)			1644.1 (1394.2–1927.6)		1515.4 (1235.4–1788.9)		-
Nutrients with DG										
Protein, % energy	15.0 (13.1–17.1)	30.7	15.2 (13.4–17.1)	26.6		15.0 (13.1–17.2)	31.7	14.7 (12.6–17.0)	36.6	<0.001
		(7.0)		(6.0)			(7.8)		(7.9)	
Fat, % energy	27.2 (22.3–32.3)	51.1	28.4 (23.8–33.1)	51.0		26.8 (22.132.0)	50.4	25.4 (20.2–30.9)	52.0	0.44
		(35.1)		(40.7)			(33.1)		(27.7)	
Saturated fat, % energy	7.1 (5.4–9.1)	51.9	8.1 (6.5–9.9)	67.1		6.8 (5.1–8.6)	46.0	5.8 (4.3–7.6)	32.2	<0.001
Carbohydrate, % energy	57.3 (51.8–63.1)	36.3	56.2 (50.9–61.4)	33.4		57.7 (52.1–63.3)	36.2	59.4 (53.3–65.8)	41.3	<0.001
		(17.9)		(12.1)			(18.5)		(27.3)	
Dietary fibre, g/day	15.5 (12.0–19.5)	64.8	15.6 (12.2–19.3)	65.2		15.9 (12.2–20.0)	62.4	15.1 (11.6–19.4)	66.4	0.05
Sodium (salt-equivalent), g/day	10.0 (7.9–12.4)	85.2	9.6 (7.8–11.8)	83.8		10.2 (8.1–12.5)	86.3	10.5 (8.2–13.2)	86.3	0.001
Potassium, mg/day	2405 (1965–2911)	60.9	2513 (2099–2989)	55.4		2429 (1989–2944)	59.5	2190 (1771–2676)	71.8	<0.001
Nutrients with EAR										
Protein, g/day	70.6 (61.7–80.6)	0.9	71.5 (62.9–80.6)	0.4		70.4 (61.7–80.6)	0.7	69.1 (59.4–80.4)	1.8	<0.001
Vitamin A, μg RE/day	446 (292–651)	55.2	485 (339–679)	49.7		425 (282–640)	57.5	382 (227–607)	62.4	<0.001
Thiamine, mg/day	0.83 (0.67–1.03)	55.8	0.84 (0.70–1.03)	54.1		0.83 (0.66–1.04)	55.6	0.80 (0.63–1.03)	58.8	<0.001
Riboflavin, mg/day	1.17 (0.93–1.44)	28.8	1.27 (1.06–1.53)	17.3		1.14 (0.91–1.40)	31.8	1.01 (0.80–1.29)	45.4	<0.001
Niacin, NEmg/day	26.3 (22.0–31.4)	0.1	26.1 (22.2–31.0)	0.0		26.7 (22.3–31.8)	.	26.4 (21.4–32.1)	0.2	0.003
Vitamin B-6, mg/day	1.15 (0.92–1.41)	33.5	1.16 (0.94–1.41)	31.5		1.16 (0.93–1.43)	32.4	1.12 (0.88–1.39)	37.7	<0.001
Vitamin B-12, μg/day	4.2 (2.2–8.2)	21.3	4.3 (2.5–8.0)	16.3		4.3 (2.2–8.3)	22.3	4.0 (1.8–8.6)	28.7	<0.001
Folate, μg/day	299 (230–382)	15.4	297 (233–375)	14.3		306 (236–395)	13.6	293 (219–382)	19.0	<0.001
Vitamin C, mg/day	92.2 (57.9–143.1)	45.5	93.5 (60.1–143.4)	44.1		100.1 (62.3–151.7)	42.0	83.3 (48.8–132.9)	51.4	<0.001
Calcium, mg/day	521 (377–683)	52.4	618 (484–774)	34.1		501 (387–646)	56.4	373 (280–503)	79.3	<0.001
Magnesium, mg/day	254 (212–302)	38.1	258 (217–304)	34.9		258 (216–309)	36.5	242 (199–292)	45.0	<0.001
Iron, mg/day	7.9 (6.5–9.6)	29.1	7.7 (6.4–9.3)	30.6		8.2 (6.6–9.8)	25.0	8.1 (6.6–9.9)	30.6	<0.001
Zinc, mg/day	7.9 (7.0–9.0)	7.8	8.1 (7.2–9.1)	5.9		7.9 (6.9–8.9)	8.2	7.8 (6.8–8.9)	10.7	<0.001
Copper, mg/day	1.2 (1.0–1.4)	0.6	1.1 (1.0–1.3)	0.6		1.2 (1.0–1.4)	0.6	1.2 (1.0–1.4)	0.7	0.95

^1^ Intakes values are medians (IQR). Nutrients expressed as energy-adjusted values by using the following equation: energy-adjusted intake (unit/day) = observed intake (unit/day) × Estimated Energy Requirement (EER, kcal/day)/observed energy intake (kcal/day). ^2^ Prevalence of not meeting DRIs is percentage of participants whose intake fell outside the range of DG or below the EAR. Each energy-adjusted nutrient intake (unit/d) was compared with each DRI value (unit/day), using the cut-point method. The probability of inadequacy of >50% for menstruating women whose bioavailability of iron is 15% (<9.3 mg/day) was considered not meeting EAR for women aged 20–49 years. ^3^ All nutrient intakes, except vitamin B12 in men, were significantly different across the dairy intake groups (*p* < 0.05; Kruskal–Wallis test). ^4^ Milk, milk consumers; Dairy, other dairy consumers; Non-dairy, non-dairy consumers; RE, retinol equivalent; NE, niacin equivalent. ^5^ Difference in the prevalence of not meeting DG and EAR was tested using Cochran–Mantel–Haenszel test adjusting for age categories.

**Table 6:** Number of nutrients with not meeting DG and EAR among 9987 men and 11,619 women according to dairy intake status ^1,2.^

	All	Milk	Dairy	Non-Dairy	*p* ^3^
Men					
Number of nutrients with not meeting DG	3.9 ± 1.5	3.8 ± 1.5	3.8 ± 1.5	4.0 ± 1.5	<0.001
Number of nutrients with not meeting EAR	4.0 ± 2.5	3.3 ± 2.4	3.9 ± 2.5	4.7 ± 2.5	<0.001
Women					
Number of nutrients with not meeting DG	3.8 ± 1.5	3.8 ± 1.5	3.7 ± 1.5	3.9 ± 1.5	0.08
Number of nutrients with not meeting EAR	3.8 ± 2.7	3.3 ± 2.6	3.9 ± 2.6	4.7 ± 2.7	<0.001

^1^ Milk, milk consumers; Dairy, other dairy consumers; Non-dairy, non-dairy consumers. ^2^ Values are mean ± SD. ^3^ Significance of difference between groups was compared using ANCOVA adjusting for age categories.
